# Versatile Cloning Strategy for Efficient Multigene Editing in *Arabidopsis*

**DOI:** 10.21769/BioProtoc.5029

**Published:** 2024-07-05

**Authors:** Ziqiang P. Li, Jennifer Huard, Emmanuelle M. Bayer, Valérie Wattelet-Boyer

**Affiliations:** 1 UMR 5200 Laboratoire de Biogenèse Membranaire, CNRS-University of Bordeaux, Villenave d’Ornon, France; 2 Institute of Science and Technology Austria, Klosterneuburg, Austria

**Keywords:** Plant genome editing, CRISPR-Cas9, *Arabidopsis thaliana*, sgRNA multiplexing, Gateway**^®^** cloning, Golden Gate cloning

## Abstract

CRISPR-Cas9 technology has become an essential tool for plant genome editing. Recent advancements have significantly improved the ability to target multiple genes simultaneously within the same genetic background through various strategies. Additionally, there has been significant progress in developing methods for inducible or tissue-specific editing. These advancements offer numerous possibilities for tailored genome modifications. Building upon existing research, we have developed an optimized and modular strategy allowing the targeting of several genes simultaneously in combination with the synchronized expression of the Cas9 endonuclease in the egg cell. This system allows significant editing efficiency while avoiding mosaicism. In addition, the versatile system we propose allows adaptation to inducible and/or tissue-specific edition according to the promoter chosen to drive the expression of the Cas9 gene. Here, we describe a step-by-step protocol for generating the binary vector necessary for establishing *Arabidopsis* edited lines using a versatile cloning strategy that combines Gateway^®^ and Golden Gate technologies. We describe a versatile system that allows the cloning of as many guides as needed to target DNA, which can be multiplexed into a polycistronic gene and combined in the same construct with sequences for the expression of the Cas9 endonuclease. The expression of Cas9 is controlled by selecting from among a collection of promoters, including constitutive, inducible, ubiquitous, or tissue-specific promoters. Only one vector containing the polycistronic gene (tRNA-sgRNA) needs to be constructed. For that, sgRNA (composed of protospacers chosen to target the gene of interest and sgRNA scaffold) is cloned in tandem with the pre-tRNA sequence. Then, a single recombination reaction is required to assemble the promoter, the zCas9 coding sequence, and the tRNA-gRNA polycistronic gene. Each element is cloned in an entry vector and finally assembled according to the Multisite Gateway^®^ Technology. Here, we detail the process to express zCas9 under the control of egg cell promoter fused to enhancer sequence (EC1.2en-EC1.1p) and to simultaneously target two multiple C2 domains and transmembrane region protein genes (*MCTP3* and *MCTP4*, respectively at3g57880 and at1g51570), using one or two sgRNA per gene.

Key features

• A simple method for *Arabidopsis* edited lines establishment using CRISPR-Cas9 technology

• Versatile cloning strategy combining various technologies for convenient cloning (Gateway^®^, Golden Gate)

• Multigene targeting with high efficiency

## Background

CRISPR-Cas9 technology, which serves as a powerful genome editing tool, is based on the bacterial RNA-guided CRISPR-Cas9 system [1]. The editing process involves two main actors: the Cas9 nuclease and a unique guide RNA (sgRNA) that will direct the Cas9 protein to the DNA target for genome editing. Plants, like other organisms, need these elements to form the CRISPR-Cas9 complex, which will be directed to the targeted sequence depending on the crRNA part of the sgRNA [2]. Plant transformation with binary vectors containing sequences for Cas9 expression and sgRNA synthesis remains one of the primary and widely used methods for the production of these actors in cells. Numerous vectors and editing strategies have been developed, such as strategies to target multiple genes simultaneously through sgRNA multiplexing. This approach addresses challenges such as gene redundancy and enhancing editing efficiency, eliminating the need for time-consuming multiple crossings [3–6]. Other ways of improvement using various promoters have been explored to optimize the expression of the Cas9, especially in the context of multitargeting but also to avoid mosaic mutations. Indeed, while promoter UBQ10 enhances mutation efficiency in comparison to the 35S promoter, ubiquitous promoters driving the expression of Cas9 could lead to mosaic mutation patterns [7]. To fix this problem, egg cell–specific promoters like EC1.2, embryo sac, embryo, and endosperm, and pollen-specific promoters like YAO or NUC1 can be used. In these conditions, Cas9 expression induces homozygous or biallelic mutants in the early generations removing the problem of mosaicism [8–10]. Taking advantage of previously published works, we chose efficient elements and strategies and merged them to develop a very versatile cloning system that combines MultiSite Gateway^®^ [11] and Golden Gate [12] technologies.

**Figure 1.**
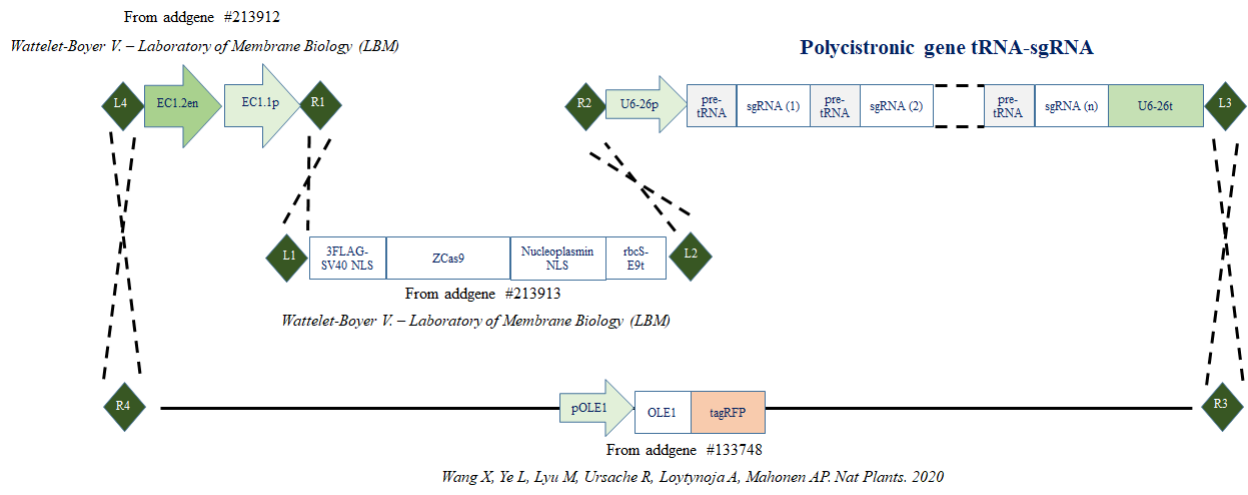
Cloning strategy. Cloning strategy to express zCas9 under the control of egg cell promoter (EC1.1p) fused to enhancer sequence (EC1.2en) and to target DNA using several sgRNA cloned in the form of a polycistronic gene. (R1, R2, R3, R4, L1, L2, L3 and L4 represent recombination sites.)

In this protocol, we describe the step-by-step procedure for generating the binary vector that contains the endogenous tRNA-processing system, previously described to boost the targeting and multiplex editing capability of the CRISPR/Cas9 system [4], and the egg cell–specific promoter EC1.2 associated to EC1.1 enhancer to drive the expression of Cas9 [8] (Figure 1). For precise genome editing, this protocol can be readily adjusted to accommodate various designs of binary vectors. This includes the utilization of different tissue-specific promoters, inducible promoters, or alternative nucleases such as SaCas9, Cas12a, or zCas9i, which can enhance both the efficiency and specificity of editing [13–17].

## Materials and reagents

**Biological materials**

1. pHEE401E plasmid (Addgene, catalog number: 71287)
2. pGTR plasmid (Addgene, catalog number: 63143)
3. pFRm43GW plasmid (Addgene, catalog number: 133748)
4. p4P1R_EC1.2en-EC1.1p plasmid (Addgene, catalog number: 213912)
5. pDONR221_zCas9 plasmid (Addgene, catalog number: 213913)
6. pDONR^TM^ P2R-P3 (Thermo Fisher Scientific, catalog number: 12537-023)
7. One Shot^TM^ TOP10 chemically competent *E. coli* (Thermo Fisher Scientific, catalog number: C404010)

**List of primers common to all strategies**

Order common and specific primers according to the following features:

Synthesis scale: 0.05 μmol; purification: cartridge; format: in solution (water); concentration: 100 μM.

(Supplier: Merck, but could be another one)

*Fw-Primer 1: 5′-* GGGGACAGCTTTCTTGTACAAAGTGGAACGACTTGCCTTCCGCACAATAC *- 3*′

*Rev-Primer 2: 5′- atggtctca*TGTTAATCACTACTTCGACTCTAG *- 3*′

*Fw-Primer 3: 5′- taggtctcc*AACAAAGCACCAGTGGTCTAGTGG *- 3*′

*Rev-Primer 12: 5′- atggtctca*AAGCACCGACTCGGTGCCACTTTTTC *- 3*′

*Fw-Primer 13: 5′- taggtctcc*GCTTTTTTTTGCAAAATTTTCCAGTCG *- 3*′

*Rev-Primer 14: 5′-* GGGGACAACTTTGTATAATAAAGTTGATATTGGTTTATCTCATCGGAAC *- 3*′

*M13-Fw (-20): 5′-* GTAAAACGACGGCCAG *- 3*′

*M13-Rev: 5′-* CAGGAAACAGCTATGAC *- 3*′

**Reagents**

**Enzymes for molecular biology**

1. Q5^®^ high-fidelity DNA polymerase (NEB, catalog number: M0491)
2. One*Taq* 2× Master Mix with standard buffer (NEB, catalog number: M0482)
3. BsaI-HF restriction enzyme (NEB, catalog number: R3733)
4. T4 DNA ligase (Thermo Fisher Scientific, catalog number: 15224025)
5. Gateway^TM^ BP Clonase^TM^ II (Thermo Fisher Scientific, catalog number: 11789020)
6. Gateway^TM^ LR Clonase^TM^ II (Thermo Fisher Scientific, catalog number: 11791020)

**Reagents for molecular biology**

1. Cutsmart buffer (NEB, catalog number: B6004)
2. dNTP solution MIX 10 mM (NEB, catalog number: N0447)
3. TAE (Tris-Acetate-EDTA buffer) (Euromedex, catalog number: EU0202)
4. SYBR^TM^ Safe DNA gel stain (Thermo Fisher Scientific, catalog number: S33102)
5. Agarose (Euromedex, catalog number: LE-8200)
6. DNase-free water (autoclaved at 110 °C for 30 min)

**Reagents for microbiology**

1. LB Miller (Euromedex, catalog number: AE-0103)
2. Bacteriological agar (Euromedex, catalog number: 1330)
3. Kanamycin bisulfate (Euromedex, catalog number: EU0420)
4. Spectinomycin dihydrochloride pentahydrate (Merck, catalog number: S4014)

**Kit for molecular biology**

1. Monarch^®^ PCR & DNA Cleanup kit (NEB, catalog number: T1030)
2. NucleoSpin^®^ Plasmid kit (Macherey Nagel, catalog number: 740588)

**Solutions**

1. Kanamycin bisulfate stock solution (25 mg/mL) (see Recipes)
2. Spectinomycin dihydrochloride pentahydrate stock solution (50 mg/mL) (see Recipes)
3. LB Miller media (see Recipes)

**Recipes**

1. **Kanamycin bisulfate stock solution (25 mg/mL)**
  **Table:** 

Reagent	Final concentration	Amount
Kanamycin bisulfate	25 mg/mL	0.25 g
ddH_2_O	n/a	10 mL
Total	n/a	10 mL
  Filter with a 0.22 μm filter, aliquot by 1 mL, and store at -20 °C.
2. **Spectinomycin dihydrochloride pentahydrate stock solution (50 mg/mL)**
  **Table:** 

Reagent	Final concentration	Amount
Spectinomycin dihydrochloride pentahydrate	50 mg/mL	0.5 g
ddH_2_O	n/a	10 mL
Total	n/a	10 mL
  Filter with a 0.22 μm filter, aliquot by 1 mL, and store at -20 °C.
3. **LB Miller media**
  **Table:** 

Reagent	Final concentration	Amount
LB Miller	25 g/L	25 g
Bacteriological agar	20 g/L	20 g
ddH_2_O	n/a	1,000 mL
Total	n/a	1,000 mL
Autoclave at 110 °C for 30 min
  Wait until the temperature of the media is at approximately 60 °C and add the antibiotic (final concentration: kanamycin bisulfate 25 μg/mL or spectinomycin dihydrochloride pentahydrate 50 μg/mL).

**Laboratory supplies**

1. Reaction tubes, 0.5 mL, PP (SARSTEDT, catalog number: 72699)
2. Reaction tubes, 1.5 mL, PP (SARSTEDT, catalog number: 72690001)
3. Petri dishes, Ø 90 mm (VWR, catalog number: 391-0556)
4. Wooden toothpicks length 80 mm (DUTSCHER, catalog number: 505802)
5. Glass beads, Ø 5 mm (DUTSCHER, catalog number: 068503)
6. 10/20 μL XL graduated TipOne^®^ tips (STARLAB, catalog number: S1110-3700-C)
7. 200 μL UltraPoint® graduated TipOne^®^ tips (STARLAB, catalog number: S1113-1700-C)
8. 1250 μL XL graduated TipOne^®^ tips (STARLAB, catalog number: S1112-1720-C)
9. Microtubes 0.2 mL + flat caps (DUTSCHER, catalog number: 010208)

## Equipment

1. Thermocycler (BIORAD, C1000 Touch Thermal Cycler, catalog number: 1851148)
2. Complete Vortex Genie 2 (DOMINIQUE DUTSCHER, catalog number: 079008)
3. Electrophoresis chamber Mupid ONE (DOMINIQUE DUTSCHER, catalog number: 088900)
4. Water bath (VWR, catalog number: IKAA20004382)
5. Shaking incubator, digital, benchtop, ES-20 (VWR, catalog number: 444-0936)
6. Oven (DOMINIQUE DUTSCHER, catalog number: 485134)
7. NanoDrop 2000 (THERMOFISHER SCIENTIFIC, catalog number: ND-2000)

## Software and datasets

1. Web application tool CRISPR-P (v1.0, 2014) (http://crispr.hzau.edu.cn/CRISPR/) [18]

## Procedure

- **Design SgRNA sequences and primers**
  1. Use the web application tool CRISPR-P to choose sgRNA (http://crispr.hzau.edu.cn/CRISPR/).
  2. Select *start design* and choose the target genome [*Arabidopsis thaliana* (TAIR10)].
  3. Enter the locus tag (e.g., *At3g57880* for At*MCTP3*).
  4. Choose a sequence guide (protospacer) according to several criteria:
    - Choose the best location to target your gene of interest. Refer to the sgRNA target on the gene map (highlighted in yellow, Figure 2a).
    - The guide score must be as close to 100% as possible (the higher the score, the more effective and specific the sgRNA is) (Figure 2b); we recommend a 98% cutoff guide score value for an effective and specific sgRNA.
    - Avoid sgRNA with possible off-target effects; if this is not possible, proscribe the potential off-target in exon (refer to the number of off-target sites, Figure 2c).
    - Identify the 20-nucleotide sequence of the selected protospacer [refer to guide sequence, Figure 2c; the PAM (protospacer adjacent motif) sequence is highlighted in green].
    **Figure 2.**
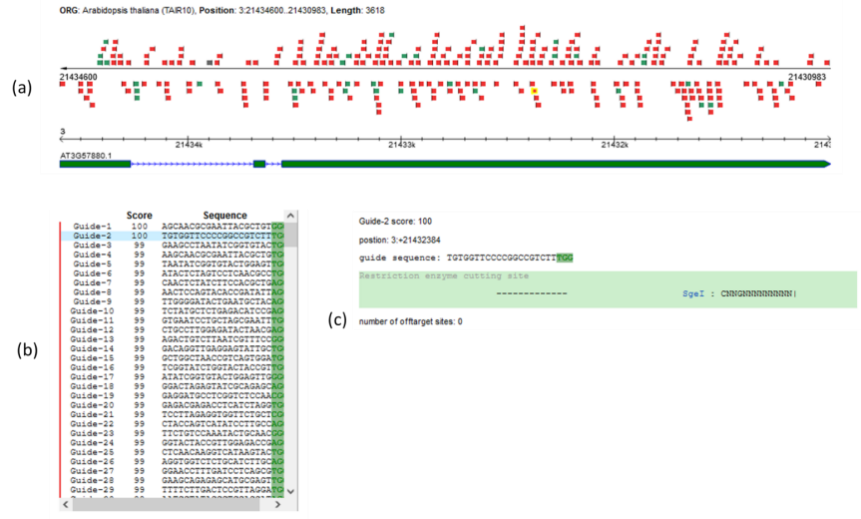
CRISPR sgRNA design using the web application tool CRISPR-P [18]. Use the web application tool CRISPR-P to choose the best guides according to their location (a), specificity (b), and possible off-target effects (c). Here, we present data corresponding to the protospacer used in sgRNA3 designed to target the At*MCTP3* gene, described in Appendix 1.
- **Construction of the polycistronic gene**
*Note: The protocol described here is inspired by the already published sgRNA multiplexing system coupled with the endogenous tRNA processing system* [4].
  The polycistronic gene is made of the same repeated modules (t-RNA, sgRNA scaffold) regularly interspaced by several guide sequences (spacers) designed with the CRISPR-P web application tool. In this protocol, we describe how to produce a molecular construction allowing the assembly of four sgRNAs (Figure 3); however, the design is the same to clone more sgRNA, and numbers of t-RNA and sgRNA scaffold modules just have to be implemented.
  **Figure 3.**
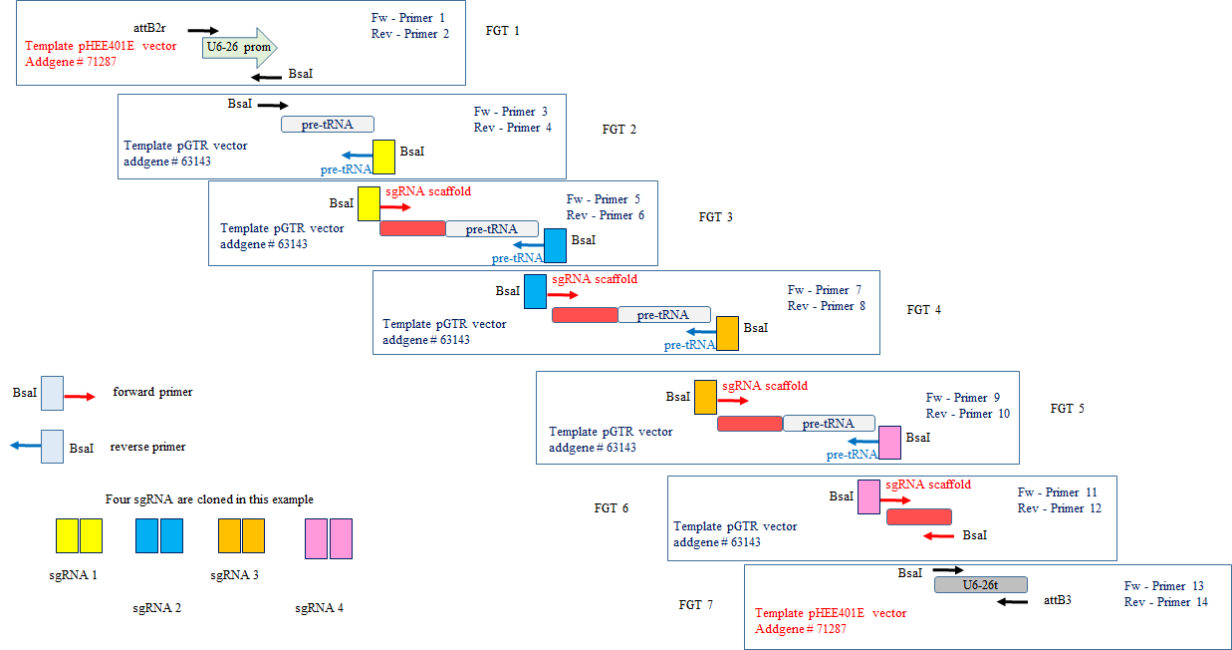
CRISPR sgRNA multiplexing in tRNA-sgRNA polycistronic gene. Multiple PCR reactions are necessary to obtain all the elements of the polycistronic gene (promoter, fusions of tRNA-guide-sgRNA scaffold, terminator). After purification, all the elements are assembled by digestion/ligation according to the Golden Gate cloning method to form the polycistronic gene, which is then ready to clone in entry vector pDONR^TM^ P2R-P3 using Gateway^®^ Technology.
  1. Primers design
    All modules are assembled according to the Golden Gate cloning system. Spacers are the only unique sequences in the polycistronic gene. Their sequences are used to design primers containing overhang extremities to perform the Golden Gate reaction (Figure 4; refer also to [4] Sup. Data Figure S6). In addition, the final Golden Gate reaction product is then cloned in an acceptor vector according to the Gateway^®^ Technology Cloning. For that, recombination sequences are required at the extremities.
    See Appendix 2 for details of amplified elements and primer sequences required for the assembly of the polycistronic gene targeting At*MCTP3*, At*MCTP4*.
*Note: The primer design method described here is from the previously published sgRNA multiplexing system coupled with the endogenous tRNA processing system (Xie et al., PNAS 2015, Sup. Data Figure S6). Adaptations were made to include the AtU6-26 promoter, U6-26 terminator, and attB sequences at the ends of the polycistronic gene for recombination into the entry vector pDONR^TM^ P2R-P3.*
    **Figure 4.**
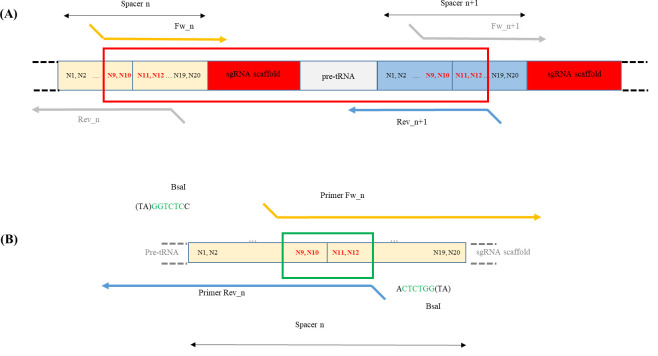
Primers design and example of PCR product. A. The polycistronic gene consists of a succession of pre-tRNA sequences, guide sequences used for targeting, and gRNA scaffolds. The guide sequences used for the Golden Gate cloning multiplexing strategy have to be included in the primers allowing the amplification of the sgRNA scaffold and pre-tRNA modules. An example of an amplification product is shown in the red box. B. Structure of the forward and reverse primers designed for the construction of a guide sequence (spacer n). A BsaI site is added at 5' of each primer for Golden Gate cloning. Nucleotides 9–12 of the guide sequence will serve as the sticky end after digestion with BsaI (green box). The 3' end of the forward primer is complementary to the start of the sgRNA scaffold, while the 3' end of the reverse primer is inversely complementary to the end of the pre-tRNA module.
    Part of the primers shown in Figure 3 will be common to all editing strategies; see below:
    (attB2r and attB3 recombination sites: bold; BsaI restriction sites: underline; overhang site for Golden Gate cloning: italics)
*Fw-Primer 1: 5′-* **GGGGACAGCTTTCTTGTACAAAGTGGAA**CGACTTGCCTTCCGCACAATAC - 3′
*Rev-Primer 2: 5′-* atggtctca *TGTT* AATCACTACTTCGACTCTAG - 3′
*Fw-Primer 3: 5′-* taggtctcc *AACA* AAGCACCAGTGGTCTAGTGG - 3′
*Rev-Primer 12: 5′-* atggtctca *AAGC* ACCGACTCGGTGCCACTTTTTC - 3′
*Fw-Primer 13: 5′-* taggtctcc *GCTT* TTTTTTGCAAAATTTTCCAGTCG - 3′
*Rev-Primer 14: 5′-* **GGGGACAACTTTGTATAATAAAGTTGA**TATTGGTTTATCTCATCGGAAC - 3′
    The other primers (from 4 to 11 in this example, Figure 3), which are necessary for the synthesis of the guide sequence, must be designed according to the indications below:
*(N1 to N20 correspond to the 20 nucleotides of the gRNA spacer)*
*Note: Be careful to avoid any guide sequence containing a BsaI restriction site. This would make polycistronic gene assembly impossible.*
*Forward primer: Fw_n, containing BsaI restriction site and the second half part of the guide sequence [nucleotides 9 to 20 (forward sequence)] fused to the 5′ end sequence of the gRNA scaffold (forward sequence).*
    5′- taggtctcn-N9N10N11N12N13N14N15N16N17N18N19N20-gttttagagctagaa-3′
*Reverse primer: Rev_n, containing BsaI restriction site and the first half part of the guide sequence [nucleotides 1 to 12 (reverse complement sequence)] fused to the 3′ end sequence of the pre tRNA (reverse complement sequence).*
    5′- cgggtctcn-N12N11N10N9N8N7N6N5N4N3N2N1-tgcaccagccggg-3′
  2. Cloning of the polycistronic gene
    The polycistronic gene will be synthesized by assembling various PCR products whose ends are made cohesive after digestion. The assembly of these modules is done in an orderly manner according to the cohesive ends designed for the Golden Gate cloning strategy.
    The steps consist of the amplification of each module by PCR, the purification of the PCR products, the assembly by a succession of digestions and ligations according to the Golden Gate cloning method, and then the cloning into an entry vector using Gateway^®^ Technology.
    These steps are detailed below. We take the example of a molecular construction allowing the assembly of 4 sgRNAs (Figure 3).
    - Amplification of each module by PCR (See Table 1 for the list of modules that will be amplified, refer to Table 2 and Table 3 for the PCR protocol and program).
      **Table 1.** List of modules to amplify . For each module, the template, the primers, and the size of the PCR product are detailed. See Appendix 2 for the sequence of amplified elements and primers used.

Module ID	Template	Forward primer	Reverse primer	PCR product size
U6-26 promoter	pHEE401E (Addgene, catalog number: 71287)	Fw-Primer1	Rev-Primer2	465 bp
Pre-tRNA(1)	pGTR (Addgene, catalog number: 63143)	Fw-Primer3	Rev-Primer4	107 bp
sgRNA(1)-pre-tRNA(2)	pGTR (Addgene, catalog number: 63143)	Fw-Primer5	Rev-Primer6	195 bp
sgRNA(2)-pre-tRNA(3)	pGTR (Addgene, catalog number: 63143)	Fw-Primer7	Rev-Primer8	195 bp
sgRNA(3pre-tRNA(4)	pGTR (Addgene, catalog number: 63143)	Fw-Primer9	Rev-Primer10	195 bp
sgRNA(4)	pGTR (Addgene, catalog number: 63143)	Fw-Primer11	Rev-Primer12	108 bp
U6-26 terminator	pHEE401E (Addgene, catalog number: 71287)	Fw-Primer13	Rev-Primer14	230 bp
**Material needed**
      10 μM diluted primers
      Q5^®^ High-Fidelity DNA Polymerase
      dNTP solution MIX 10 mM
      pHEE401E plasmid
      pGTR plasmid
      DNase-free water
      Agarose
      TAE (Tris-Acetate-EDTA buffer)
      SYBR^TM^ Safe DNA gel stain
      Monarch^®^ PCR & DNA Cleanup kit
      **Table 2.** PCR protocol for one reaction

Components	Volume
Template (4 ng/µL)	1 μL
5× Q5 reaction buffer	10 μL
dNTP (10 mM)	1 μL
Forward primer (10 μM)	1 μL
Reverse primer (10 μM)	1 μL
Q5 DNA polymerase	0.5 μL
DNase-free water	35.5 μL
      **Table 3.** PCR program (inspired by NEB’s instructions)

Temperature	Time	Nb of cycles
98 °C	30 s	1×
98 °C	10 s	25×
TM °C	30 s	
72 °C	1 min/kb	
72 °C	2 min	1×
12 °C	Hold
      Troubleshooting: These reaction conditions are optimal, but if despite everything no amplification is obtained, it is recommended to use new solutions (buffer, dNTP), to re-dilute the primers, and to use a new aliquot of DNase-free water.
    - PCR product purification
      i. 5 μL of each PCR product is checked by electrophoresis on agarose gel (1%) – TAE 0.05% – SYBR^TM^ Safe 0.005%.
      ii. If the amplification is specific and leads to a single band on an agarose gel, the PCR products can then be purified using a suitable purification kit (e.g., Monarch^®^ PCR & DNA Cleanup kit).
      iii. Elute in 10 µL of elution buffer; samples with higher DNA yield can be diluted after PCR purification.
    - Assembly of purified PCR products using the Golden Gate cloning system
      The protocol consists of a succession of digestions and ligations of the purified PCR products in order to construct the polycistronic gene with the attB2r and attB3 recombination sites at its ends (See Table 4 and 5 for Golden Gate reaction and incubation conditions).
**Material needed**
      Purified PCR products
      BsaI-HF restriction enzyme
      10× Cutsmart buffer
      T4 DNA ligase
      DNase-free water
      **Table 4.** Golden Gate reaction

Components	Volume
Purified PCR products 1	2 μL
Purified PCR products 2	2 μL
Purified PCR products 3	2 μL
Purified PCR products 4	2 μL
Purified PCR products 5	2 μL
Purified PCR products 6	2 μL
Purified PCR products 7	2 μL
10× Cutsmart buffer	2 μL
BsaI-HF	1 μL
T4 DNA ligase	1.5 μL
DNase-free water	1.5 μL
      Use a thermocycler to perform successive digestion and ligation according to the program below:
      **Table 5.** Golden Gate incubation conditions

Temperature	Time	Nb of cycles
40 °C	10 min	3×
16 °C	10 min	
50 °C	10 min	1×
80 °C	20 min	1×
    - Cloning of the polycistronic gene into the pDONR^TM^ P2R-P3 vector
      The recombination sites flanked Golden Gate reaction product are integrated into the pDONR^TM ^P2R-P3 vector according to the Multisite Gateway^®^ Technology protocol and various recombination protein activities (Figure 5). (See Table 6 for BP reaction conditions)
*Note: Some minor modifications were made to the Multisite Gateway^®^ Technology protocol. Namely, BP recombination reaction volume is reduced from 10 to 5 μL, we do not work with an equimolar ratio between insert and entry vector but systematically add 3 μL of Golden Gate reaction product regardless of the concentration, and no Proteinase K treatment is necessary after the BP recombination reaction.*
      **Figure 5.**
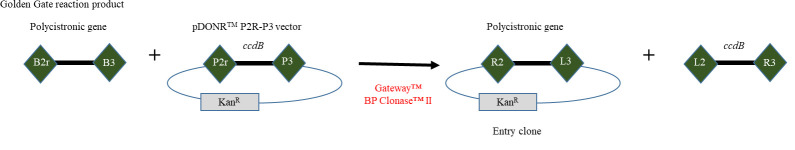
Generating the entry clone containing the polycistronic gene. Figure adapted from the user guide “MultiSite Gateway^®^ Three-Fragment Vector Construction Kit.” A BP recombination reaction is performed between the *att*B2r and *att*B3-flanked polycistronic gene and pDONR™ P2R-P3 to generate an entry clone.
**Material needed**
*att*B2r-Golden Gate reaction product-*att*B3
      Purified plasmid DNA pDONR^TM^ P2R-P3 (150 ng/μL)
      Gateway^TM^ BP Clonase^TM^ II
      One Shot^TM^ TOP10 chemically competent *E. coli*
      LB Miller media
      Kanamycin bisulfate
      Bacteriological agar
      **Table 6.** BP reaction conditions

Components	Volume
Golden Gate reaction product	3 μL
pDONR^TM^ P2R-P3	1 μL
Gateway^TM^ BP Clonase^TM^ II	1 μL

*Note: Be sure to keep the BP Clonase* ^®^ *II enzyme mix on ice while preparing the recombination reaction. Return the enzyme mix to -20 °C immediately after use.*
      Incubate overnight at 25 °C.
      The day after, use all the LR reaction to transform chemically competent cells:
      i. Add 5 μL of LR reaction to 50 μL of One Shot^TM^ TOP10 chemically competent *E. coli* (e.g.).
      ii. Incubate for 30 min on ice.
      iii. Heat shock for 1 min 30 s at 37 °C.
      iv. Transfer on ice for 2 min.
      v. Add 1 mL of LB Miller media.
      vi. Shake for 45 min at 37 °C.
      vii. Spread with glass beads on LB Miller agar plate containing kanamycin (25 µg/mL).
      viii. Incubate the plate overnight at 37 °C.
      Troubleshooting: If no colony is obtained after transformation, repeat the reaction under the same conditions. If the problem persists, this is probably due to the enzyme mixture, which may have been stored on ice for too long before being returned to -20 °C during the previous use.
    - Screening positive colonies by PCR
      Prepare a PCR reaction mix (conditions for one reaction are described in Table 7. See also Table 8 for PCR program) and use colonies as templates to check whether or not they contain the construct.
**Material needed**
      10 μM diluted primers
      Use universal primers (see below) or specific primers for the polycistronic gene:
      M13 Forward (-20): 5′-GTAAAACGACGGCCAG-3′
      M13 Reverse: 5′-CAGGAAACAGCTATGAC-3′
      One*Taq* 2× Master Mix with standard buffer
      **Table 7.** PCR Protocol

Components	Volume
One*Taq* 2× Master	12.5 μL
Forward primer (10 μM)	1 μL
Reverse primer (10 μM)	1 μL
DNase-free water	10.5 μL
      i. Use a toothpick to pick a colony and spread it on an LB Miller agar (25 g/L) with kanamycin (25 μg/mL) plate.
      ii. Directly afterward, dip the toothpick into the PCR reaction to transfer a very small number of cells (this step is critical, too many cells could inhibit the PCR reaction).
      iii. Proceed in this way for each colony to be tested
      iv. Incubate the plate overnight at 37 °C and run the PCR in the thermocycler using the following conditions:
      **Table 8.** PCR program (adapted from NEB’s instructions)

Temperature	Time	Nb of cycles
94 °C	30 s	1×
94 °C	30 s	25×
TM °C	30 s	
68 °C	1 min/kb	
68 °C	5 min	1×
12 °C	Hold
      v. 20 μL of each PCR product are checked by electrophoresis on agarose gel (1%) – TAE 0.05% – SYBR^TM^ Safe 0.005%.
      vi. Pick corresponding positive colonies on the plate with a toothpick and inoculate 5 mL of LB Miller + kanamycin (25 μg/mL) for each.
      vii. Incubate culture and shake overnight at 37 °C.
      viii. Perform plasmid extraction and purification for each clone using a suitable kit (e.g., NucleoSpin^®^ Plasmid kit, Macherey Nagel).
      ix. Elute plasmid DNA according to the supplier’s instructions.
      x. Using a spectrophotometer, determine the sample concentration (A260 nm) and check the DNA purity (A260/A280 nm ratio should be comprised between 1.8 and 2.0).
      xi. Perform sequencing using the universal primers below and verify the integrity of the polycistronic gene sequence.
      M13 Forward (-20): 5′-GTAAAACGACGGCCAG-3′
      M13 Reverse: 5′-CAGGAAACAGCTATGAC-3′
      Troubleshooting: If no amplification is obtained after PCR reaction, repeat the reaction under the same conditions but taking care to resuspend only a very small quantity of bacteria in the reaction medium because too many cells could inhibit the reaction.
- **Assembly in destination vector**
  The promoter, ZCas9, and the polycistronic gene cloned independently into entry vectors are then assembled using Gateway^®^ Cloning technology to form a final plasmid ready for plant transformation (Figure 6). All these elements, flanked by compatible recombination sequences, will assemble in an orderly manner thanks to the integrase and excisionase activity of a mixture of enzymes according to the Multisite Gateway^®^ Technology protocol (see Table 9 for LR reaction conditions).
*Note: Some minor modifications were made to the Multisite Gateway^®^ Technology protocol. Namely, LR recombination reaction volume is reduced from 10 to 5 μL, we do not take into account the molarity but the concentration of the plasmids in ng/µL, and no Proteinase K treatment is necessary after the LR recombination reaction.*
  **Figure 6.**
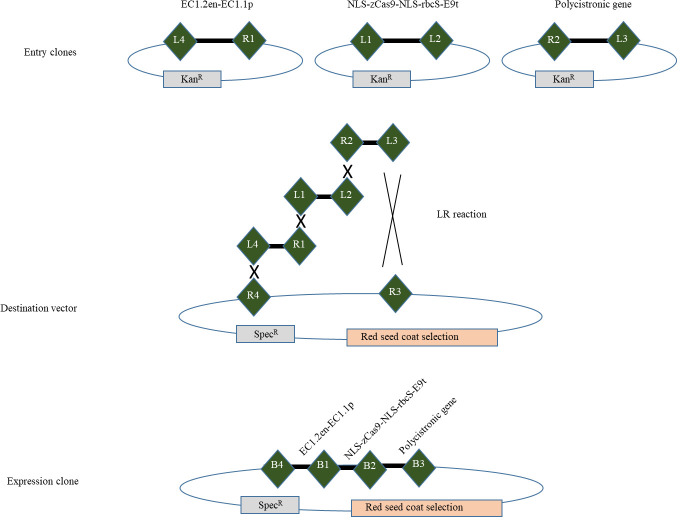
Generating the expression clone containing the promoter, zCas9, and the polycistronic gene (adapted from the user guide “MultiSite Gateway^®^ Three-Fragment Vector Construction Kit”).
  1. Homologous recombination reaction
**Material needed**
    Purified plasmid DNA of your *att*L4 and *att*R1-flanked entry clone (supercoiled, 80 ng/μL)
    Purified plasmid DNA of your *att*L1 and *att*L2-flanked entry clone (supercoiled, 80 ng/μL)
    Purified plasmid DNA of your *att*R2 and *att*L3-flanked entry clone (supercoiled, 80 ng/μL)
    Purified plasmid DNA pFRm43GW (150 ng/μL)
    Gateway^TM^ LR Clonase^TM^ II
    One Shot^TM^ TOP10 chemically competent *E. coli*
    LB Miller media
    Spectinomycin dihydrochloride pentahydrate
    Bacteriological agar
    **Table 9.** LR reaction conditions

Components	Volume
*att*L4 and *att*R1-flanked entry clone	1 μL
*att*L1 and *att*L2-flanked entry clone	1 μL
*att*R2 and *att*L3-flanked entry clone	1 μL
pFRm43GW	1 μL
Gateway^TM^ LR Clonase^TM^ II	1 μL

*Note: Be sure to keep the LR Clonase^®^ II enzyme mix on ice while preparing the recombination reaction. Return the enzyme mix to -20 °C immediately after use.*
    Incubate overnight at 25 °C.
    The day after, use all the LR reaction to transform chemically competent cells:
    i. Add 5 μL of LR reaction to 50 μL of One Shot^TM^ TOP10 chemically competent *E. coli* (e.g.).
    ii. Incubate for 30 min on ice.
    iii. Heat shock for 1 min 30 s at 37 °C.
    iv. Transfer on ice for 2 min.
    v. Add 1 mL of LB Miller media.
    vi. Shake for 45 min at 37 °C.
    vii. Spread on LB Miller agar plate containing spectinomycin (50 μg/mL).
    viii. Incubate the plate overnight at 37 °C.
    Troubleshooting: If no colony is obtained after transformation, repeat the reaction under the same conditions. If the problem persists, this is probably due to the enzyme mixture, which may have been stored on ice for too long before being returned to -20 °C during the previous use.
  2. Screening positive colonies by PCR
    Perform PCR screening as described in step B2e. Use various couples of primers to verify the integrity of the vector (Table 10); see Figure 7 for primers details.
    Troubleshooting: If no amplification is obtained after PCR reaction, repeat the reaction under the same conditions but taking care to resuspend only a very small quantity of bacteria in the reaction medium, because too many cells could inhibit the reaction.
    **Table 10.** List of primers needed for the PCR screening

**Primer ID**	**Primer sequence 5′ > 3**′	**Size of the PCR product**
EC1.2en-fw	TTGCGTTTGGTTTATCATTGCG	400 bp
EC1.2en-rv	AGTGTTGTCGATGTGTCATGT	
Zcas9-fw	GGATGATGATGACAAGATGG	450 bp
Zcas9-rv	GTGGTAGGCAACCTCGTC	
U6-26p-fw	GATTAGGCATCGAACCTTC	750 bp
U6-26t-rv	GTTTATCTCATCGGAACTGC
    **Figure 7.**
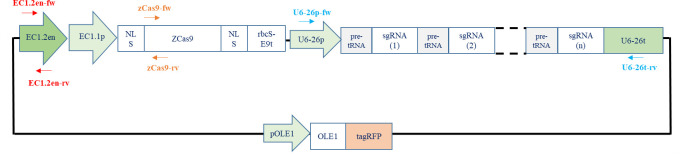
Performing PCR to check the integrity of the vector. Little arrows represent primers used to check that the promoter, zCas9, and the polycistronic gene were cloned into the destination vector.
    For colonies that were positives for the three PCR reactions:
    Perform plasmid extraction and purification as described in step B2e [use LB Miller + spectinomycin (50 µg/mL) to inoculate positive colonies in order to perform plasmid extraction and purification].
  3. Sequencing
    Perform sequencing using the primers below and verify the integrity of the destination vector (Table 11); see Figure 8 for primers details.
    **Table 11.** List of primers needed for the destination vector sequencing

**Primer ID**	**Primer sequence 5′ > 3**′
pEC1.2en-seq-F	GGAGCGCTACTGATTCAAC
Cas9Seq_658_678	GTTGACAAGCTGTTCATCCAG
Cas9Seq_1520_1539	AGTCAGAGGAGACGATCACG
Cas9Seq_2330_2349	TGCAGACCGTGAAGGTTGTG
Cas9Seq_3163_3186	TACGATGTGAGGAAGATGATCGCC
U6-26p-seq-F	TGTCCCAGGATTAGAATGATTAGGC
    **Figure 8.**
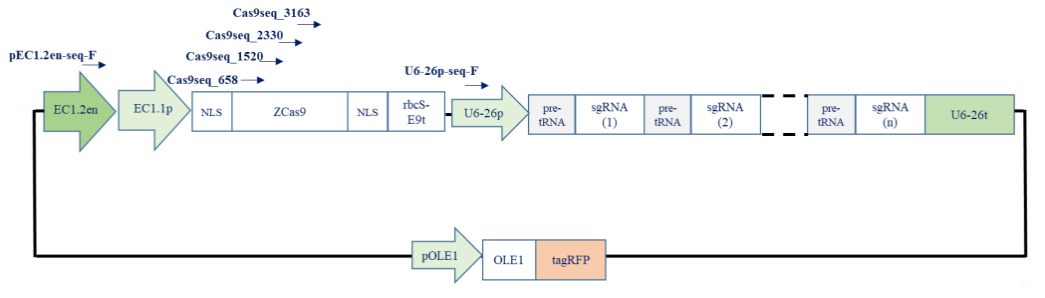
Performing sequencing to check the integrity of the vector. Little arrows represent primers used to check by sequencing that promoter, zCas9, and the polycistronic gene were assembled into the destination vector.
    After sequencing and checking, the plasmid is ready to transform *Agrobacterium tumefaciens* (strain C58C1). The transformed strain will be used for *Arabidopsis thaliana* transformation by the floral dip method [19]. Transformants are then selected according to seed tagRFP fluorescence (red seed coat selection) [20].

## Validation of protocol

At*MCTP3* and At*MCTP4* genes belong to the multiple C2 domains and transmembrane region protein (MCTP) multigenic family. Associated proteins are key regulators of cell-to-cell signaling in plants and act as ER-PM tethers specifically at plasmodesmata. The previously characterized *Atmctp3/Atmctp4* loss of function mutant induces plant developmental defects and impaired plasmodesmata function and composition [21]. This mutant is strongly affected in its development; thus, At*MCTP3* and At*MCTP4* genes are excellent candidates to validate a multi-targeting genome editing protocol.

**Figure 9.**
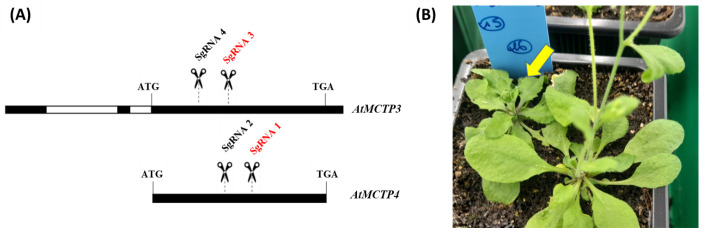
At*MCTP3* and At*MCTP4* genes targeting and associated phenotype. (A) SgRNA targeting zone in At*MCTP3* and At*MCTP4* genes. **(B)** CRISPR double mutant *Atmctp3/Atmctp4* associated phenotype (yellow arrow) compared to wild type on the side. Both black and red SgRNA were used for two guides per gene strategy. Only red guides were used for the single guide per gene strategy.

The protocol we described allowed us to edit simultaneously At*MCTP3* and At*MCTP4* genes using two guides per targeted gene and to obtain the *Atmctp3/Atmctp4* double mutant–associated phenotype (Figure 9B). We compare also with the same cloning strategy using only one guide per targeted gene. Our strategy allowed us to obtain at least 15% of biallelic CRISPR *Atmctp3/Atmctp4* mutants using one guide per gene, and 20% CRISPR *Atmctp3/Atmctp4* mutants using two guides per gene (either homozygous or biallelic mutants depending on the guide) (Table 12). Most of the time, the "two guides per gene" strategy led to big deletions for At*MCTP3* gene (around 440 bp deletion depending on the line), while only 1 or 2 bp indels were obtained for At*MCTP4* gene.

**Table 12.** Proportion of CRISPR *Atmctp3/Atmctp4* mutants for both strategies and type of mutation obtained for each sgRNA. NA = not applicable.

	*Atmctp3*, *Atmctp4* phenotype	*Atmctp3* homozygous mutation		*Atmctp3* biallelic mutation		*Atmctp4* homozygous mutation		*Atmctp4* biallelic mutation	
		**SgRNA4**	**SgRNA3**	**SgRNA4**	**SgRNA3**	**SgRNA2**	**gRNA1**	**SgRNA2**	**SgRNA1**
1 guide strategy n = 19	3/19 **15.78%**	NA	0/3	NA	3/3	NA	0/3	NA	3/3
2 guides strategy n = 24	5/24 **20.83%**	5/5	4/5	0/5	0/5	0/5	2/5	2/5	3/5

**Highlighting versatility and adaptability to inducible or cell type-specific editing**

Here, we described a cell type–specific editing system that avoids mosaicism by using the egg cell promoter to control zCas9 expression. Depending on the biological question, other cell type–specific editing strategies may be necessary (e.g., if one wants to target only the quiescent center, cortex in the root). These questions can be assessed using cell type–specific promoters. These promoters can also be used in an inducible version in association with the XVE module to allow induction with β-estradiol. To do this, the promoter of interest must be cloned in pDONR^TM^ P4-P1R vector according to Thermo Fisher’s recommendations.

Briefly, the promoter should be amplified with primers containing *attB4* and *attB1r* recombination sites (see below for primer sequences). The PCR product is then cloned into the entry clone via a BP recombination reaction (with the same conditions as described in section B2d for cloning the polycistronic gene into pDONR^TM^ P2R-P3 vector). Classical steps of *E. coli* transformation, screening, and sequencing are carried out as described previously.

Primer sequences for promoter amplification:

attB4 5’ - GGGG-ACA-ACT-TTG-TAT-**AGA-AAA**-GTT-GNN--(template-specific sequence)-3’

attB1r 5’ – GGGG-AC-TGC-**TTT-TTT**-GTA-CAA-ACT-TGN--(template-specific sequence)-3’

Finally, the newly designed vector is used for final LR recombination in association with the *att*L1 and *att*L2-flanked entry clone (encoding for the Cas9) and the *att*R2 and *att*L3-flanked entry clone (encoding for the polycistronic gene pre-tRNA/sgRNA) (following the conditions described previously).

## Supplementary information

The following supporting information can be downloaded here:

1. Appendix 1. List of protospacers chosen for At*MCTP4* and At*MCTP3* genes targeting.
2. Appendix 2. Details of amplified elements required for the polycistronic gene assembly.
